# Diagnostic Methods for Muscle Wasting in COPD: Strengths, Limitations and the Role of Nutritional Assessment

**DOI:** 10.3390/nu18203440

**Published:** 2026-10-20

**Authors:** Roberta Cianci, Domenica Francesca Mariniello, Giuseppe Luciano, Filippo Scialò, Luigi Aronne, Grazia Mazzeo, Alfredo Cipriano, Susan F. Campbell, Fabio Perrotta, Andrea Bianco, Raffaella Pagliaro

**Affiliations:** 1 Department of Translational Medical Sciences, University of Campania “Luigi Vanvitelli”, 80131 Naples, Italy; cianciroberta@libero.it (R.C.); domenica.mariniello@ospedalideicolli.it (D.F.M.); giuseppe.luciano1@studenti.unicampania.it (G.L.); luigi.aronne@gmail.com (L.A.); grazia.mazzeo@ospedalideicolli.it (G.M.); fabio.perrotta@unicampania.it (F.P.); andrea.bianco@unicampania.it (A.B.); raffaella.pagliaro@studenti.unicampania.it (R.P.); 2 U.O.C. Clinica Pneumologica L. Vanvitelli, A.O. dei Colli, Monaldi Hospital, Via Leonardo Bianchi, 80131 Naples, Italy; 3 Department of Molecular Medicine and Medical Biotechnologies, University of Naples “Federico II”, 80131 Naples, Italy; alfredo.cipriano@unina.it; 4 Department of Advanced Medical and Surgical Sciences, University of Campania “Luigi Vanvitelli”, 80138 Naples, Italy; sfmcampbell@gmail.com

**Keywords:** sarcopenia, COPD, muscle wasting, diagnostic methods, muscle mass assessment, physical performance

## Abstract

**Background/Objectives:** Skeletal muscle wasting is a frequent and clinically consequential comorbidity in COPD, affecting 15–50% of patients and predicting reduced exercise tolerance, impaired quality of life, hospitalization, and mortality. However, no consensus exists on the optimal diagnostic approach, and the role of nutritional assessment remains underemphasized despite its contribution to muscle depletion. **Methods:** This narrative review synthesizes evidence from original research, systematic reviews, and consensus statements on diagnostic methods for evaluating muscle mass, strength, physical performance, and nutritional status in COPD. **Results:** Computed tomography and magnetic resonance imaging offer accurate assessment of muscle mass but are limited by cost and accessibility. Dual-energy X-ray absorptiometry provides a low-radiation alternative but cannot assess muscle quality. Bioelectrical impedance analysis and ultrasound are portable and inexpensive, yet lack standardized COPD-specific cut-offs. Functional tests provide clinically meaningful performance data but do not directly measure muscle mass. In parallel, nutritional screening tools and dietary assessment can identify modifiable risk factors and are associated with sarcopenia prevalence and prognosis in COPD. The choice of diagnostic approach significantly influences prevalence estimates. **Conclusions:** Optimal assessment of muscle wasting in COPD requires a multimodal approach combining muscle mass quantification, strength measurement, physical performance evaluation, and nutritional assessment. Standardized COPD-specific diagnostic criteria are needed. A practical algorithm integrating simple screening tools with targeted confirmatory assessments, including nutritional evaluation, may help guide clinical practice.

## 1. Introduction

Chronic obstructive pulmonary disease (COPD) is a progressive respiratory disorder characterized by persistent airflow limitation and chronic airway inflammation [1]. Beyond its pulmonary manifestations, COPD is increasingly recognized as a multisystem disease, with skeletal muscle dysfunction representing one of the most clinically significant extrapulmonary complications [2,3]. Skeletal muscle wasting, encompassing reductions in muscle mass, strength, and physical performance, affects a substantial proportion of patients with COPD and independently predicts adverse clinical outcomes, including reduced exercise tolerance, impaired quality of life, increased healthcare utilization, and mortality [4,5,6]. The prevalence of muscle wasting in COPD is considerably higher than in the aging population, with estimates ranging from 15% to more than 50%, depending on the diagnostic criteria applied and the population studied [5,7]. This wide variability reflects both differences across patient cohorts and the lack of a universally accepted diagnostic framework for assessing muscle depletion in this setting.

The pathophysiology of muscle loss in COPD is multifactorial, involving systemic inflammation, oxidative stress, chronic hypoxia, corticosteroid exposure, physical inactivity, and nutritional deficits [2,4]. These interconnected mechanisms promote protein degradation, impair protein synthesis, and may contribute to anabolic resistance, ultimately favoring progressive skeletal muscle depletion [8]. The clinical importance of muscle wasting in COPD has driven considerable research interest in diagnostic methods. However, the optimal approach for identifying and quantifying muscle depletion in this population remains controversial. Currently, several techniques can evaluate distinct components of muscle health: imaging and bioelectrical methods for mass quantification, dynamometry for strength measurement, and standardized tests for functional performance [9]. Each modality offers a different balance of advantages and limitations in terms of accuracy, accessibility, cost, radiation exposure, and clinical feasibility.

Moreover, the definition of sarcopenia—the age-related loss of muscle mass and function—has undergone substantial evolution over the past decade, with multiple consensus panels proposing differing criteria and cut-off values [10,11,12].

This review aims to provide a comprehensive overview of current diagnostic methods for muscle wasting assessment in COPD, critically evaluating their strengths and limitations. By synthesizing evidence from consensus statements, original research, and systematic reviews, we seek to inform clinical practice and guide future research toward standardized and practical approaches for identifying and monitoring muscle depletion in this high-risk population.

## 2. Defining and Diagnosing Sarcopenia

Sarcopenia, first described in 1989, has evolved from an age-related physiological decline to a recognized muscle disease associated with disability, falls, frailty, and mortality [10,13,14]. Despite increasing recognition, no universally accepted operational definition exists. Its prevalence rises markedly with age, affecting approximately 30% of individuals > 60 years and > 50% of those > 80 years [15]. Sarcopenia is now considered a multifactorial condition arising from the interaction of ageing, chronic inflammation, endocrine and mitochondrial dysfunction, reduced physical activity, inadequate nutrition, and chronic disease [16].

Muscle wasting in chronic disease is often described as cachexia. Although distinct from sarcopenia, both conditions share mechanisms including systemic inflammation, insulin resistance, oxidative stress, and increased proteolysis [17,18]. Sarcopenia is primarily defined by reduced muscle strength and muscle quantity or quality, whereas cachexia is a systemic disease-associated wasting syndrome characterized by involuntary weight loss, muscle depletion, and often adipose tissue loss, together with inflammatory and metabolic alterations. In COPD, the two conditions may coexist and should therefore be distinguished using a multidimensional assessment that considers longitudinal changes in body weight, body composition, muscle strength, physical performance, nutritional intake, and systemic inflammation. Unlike cachexia, sarcopenia does not require weight loss, low BMI, anorexia, or systemic inflammation for diagnosis [18,19].

In COPD, distinguishing primary from secondary sarcopenia is similarly challenging because ageing and disease-related factors frequently overlap. According to EWGSOP2, primary sarcopenia is predominantly attributable to ageing, whereas secondary sarcopenia results from additional factors such as physical inactivity, inadequate nutritional intake, hypoxemia, or corticosteroid exposure [10]. Ageing also promotes anabolic resistance, characterized by a blunted muscle protein-synthetic response to amino acids; adequate protein intake, particularly leucine-rich sources and appropriate protein distribution, may help support muscle anabolism [20,21,22]. In COPD, sarcopenia is associated with reduced exercise tolerance, respiratory dysfunction, impaired quality of life, hospitalization, and mortality [2,5,23], supporting an integrated management strategy combining nutritional support, pulmonary rehabilitation, and resistance exercise [22,23,24]. Early identification is therefore important to limit mobility impairment, falls, disability, and loss of independence [25].

Several consensus frameworks have been developed to standardize sarcopenia diagnosis (Table 1). The 2010 European Working Group on Sarcopenia in Older People (EWGSOP) defined sarcopenia as low muscle mass accompanied by impaired muscle function, reflected by reduced strength or physical performance [14]. It distinguished pre-sarcopenia, sarcopenia, and severe sarcopenia according to the progressive presence of these abnormalities. Proposed thresholds included DXA-derived appendicular skeletal muscle index (ASMI) < 7.26 kg/m^2^ in men and <5.5 kg/m^2^ in women, handgrip strength < 30 and <20 kg, respectively, and an SPPB score ≤ 8; gait speed < 0.8 m/s was also proposed as a practical indicator of impaired performance [26].

The 2019 EWGSOP2 revision shifted the emphasis toward muscle strength, defining low strength as the primary indicator of probable sarcopenia [10]. Sarcopenia is considered probable when low strength is present, confirmed when reduced muscle quantity or quality is additionally demonstrated, and severe when poor physical performance is also evident. This approach reflects evidence that functional impairment may predict adverse outcomes more effectively than muscle mass alone [10].

Other groups have proposed alternative criteria. The International Working Group on Sarcopenia (IWGS) combined low muscle mass with impaired physical performance, using gait speed <1.0 m/s and low appendicular lean mass by DXA [26]. The Asian Working Group for Sarcopenia (AWGS) adapted diagnostic thresholds to Asian populations; its 2019 criteria include handgrip strength <28 kg in men and <18 kg in women, gait speed < 1.0 m/s, and DXA-derived muscle mass indices <7.0 and <5.4 kg/m^2^, respectively [12]. AWGS also introduced “possible sarcopenia” for community screening based on calf circumference and SARC-F or SARC-CalF questionnaires [12]. The Foundation for the National Institutes of Health (FNIH) Sarcopenia Project proposed adjusting appendicular lean mass for BMI rather than height squared, potentially improving the identification of sarcopenic obesity [27]. Conversely, the Sarcopenia Definition and Outcomes Consortium (SDOC) proposed a function-oriented definition based solely on low muscle strength and slow gait speed, with thresholds of <35.5 kg in men and <20 kg in women for handgrip strength and <0.8 m/s for gait speed [11].

These differing frameworks reflect ongoing debate regarding the relative importance of muscle mass, strength, and physical performance. Their methodological differences substantially affect prevalence estimates, case identification, and potentially therapeutic approaches. Future research should determine whether function-oriented models provide superior prognostic accuracy and clarify how nutritional and rehabilitative interventions can be optimally integrated into sarcopenia management.

## 3. Sarcopenia Prevalence in COPD

Patients with COPD frequently exhibit reductions in skeletal muscle mass, strength, and physical performance due to systemic inflammation, oxidative stress, chronic hypoxaemia, mitochondrial dysfunction, endocrine alterations, corticosteroid exposure, recurrent exacerbations, nutritional impairment, and physical inactivity [2,5,20]. Systemic inflammation, particularly elevated TNF-α, IL-6, and C-reactive protein, promotes proteolysis and impairs muscle protein synthesis, while oxidative stress and hypoxia further contribute to mitochondrial dysfunction and muscle degradation [28,29]. Nutritional impairment is also common in advanced COPD, reflecting increased energy expenditure, dyspnoea-related reductions in food intake, fatigue during meals, depression, and medication-related anorexia [30]. Inadequate energy and protein intake, together with age-related anabolic resistance, may accelerate muscle loss [8], whereas higher protein intake, particularly leucine-rich sources combined with exercise and pulmonary rehabilitation, may attenuate muscle depletion [21,22,23]. Muscle loss in COPD affects both peripheral and respiratory muscles and is associated with reduced exercise capacity, ventilatory dysfunction, poorer quality of life, hospitalization, and mortality [31,32]. Sarcopenia prevalence is considerably higher in COPD than in the general older population, with reported estimates ranging from approximately 15% to >50%, reflecting differences in disease severity, population characteristics, nutritional status, and diagnostic criteria [2,5]. A meta-analysis by Benz et al. estimated an overall prevalence of approximately 21.6%, while other studies have reported lower or higher rates depending on the diagnostic framework and clinical setting [5]. Sarcopenia becomes more frequent with increasing COPD severity and is particularly prevalent among patients with severe airflow limitation, recurrent exacerbations, chronic hypoxaemia, and reduced exercise tolerance [5]. Sarcopenic patients generally exhibit lower quadriceps strength, reduced walking capacity, greater frailty, and impaired daily functioning [7]. Importantly, anabolic resistance may precede measurable muscle loss, making early identification challenging. However, anabolic resistance is not currently incorporated into validated diagnostic algorithms and should therefore be regarded as a potential underlying or early biological mechanism rather than an established diagnostic phenotype. Both EWGSOP2 and AWGS 2019 consider low muscle strength in the absence of documented muscle loss as indicative of probable sarcopenia, providing a framework for identifying functional impairment before overt muscle depletion [5,10,12]. In stable COPD, metabolic studies have similarly demonstrated muscle weakness despite preserved muscle mass, accompanied by increased myofibrillar protein breakdown and metabolic abnormalities associated with impaired oxygenation [33]. The GLIM framework may complement these approaches by defining malnutrition through at least one phenotypic criterion—weight loss, low BMI, or reduced muscle mass—and one etiological criterion, such as reduced intake/assimilation or disease burden and systemic inflammation. Thus, COPD-related inflammation is recognized as a contributor to malnutrition but is not sufficient on its own for diagnosis [34,35]. Emerging tools may further improve early muscle phenotyping. Bioelectrical impedance-derived phase angle has been associated with sarcopenia and physical fitness; CT-derived muscle area and density may provide opportunistic assessment from routinely acquired chest CT scans; and muscle ultrasound, including rectus femoris measurements and shear-wave elastography, offers a non-invasive approach to muscle assessment. However, these techniques are not yet incorporated into standard COPD sarcopenia algorithms [36,37,38,39]. A validated algorithm integrating oxygenation, inflammation, muscle quality, and anabolic responsiveness is still lacking, but combining complementary measures may facilitate earlier identification of patients at risk. Sarcopenia also has important implications for respiratory function. COPD-related hyperinflation increases the mechanical load on the respiratory pump, while respiratory muscle dysfunction reduces its capacity to compensate. The diaphragm operates at a mechanical disadvantage, increasing the work of breathing and predisposing to alveolar hypoventilation and hypercapnia when ventilatory demand exceeds respiratory pump capacity. Sarcopenia may further reduce inspiratory muscle reserve, resulting in reduced inspiratory strength and effective tidal volume and contributing to elevated PaCO_2_ and type II respiratory failure. Maximal inspiratory pressure (MIP) may therefore provide useful information regarding the risk of hypercapnia [40,41,42], particularly during exacerbations, when increased ventilatory demand and worsening muscle dysfunction can rapidly compromise respiratory function [43]. Peripheral and respiratory muscles also differ in their adaptive responses. While limb muscles, particularly the quadriceps, are strongly affected by deconditioning and muscle loss, the diaphragm undergoes chronic adaptation to increased workload, including a greater proportion of oxidative fibres, enhanced oxidative capacity, and sarcomere remodelling. These adaptations may partially preserve function but cannot fully compensate for persistent hyperinflation and increased respiratory load, particularly during exacerbations or exercise [41,44]. Consequently, the concept of “respiratory sarcopenia” has emerged, although it remains less clearly defined than systemic sarcopenia. A recent position paper recognizes respiratory muscle weakness as an important component of sarcopenia in COPD [45]. Finally, COPD-associated sarcopenia contributes to a self-reinforcing cycle of dyspnoea, physical inactivity, deconditioning, and progressive loss of muscle mass and strength, which further increases exercise-related fatigue and limits activity [46,47]. Unlike fixed airway obstruction, this muscular component is at least partially modifiable. Pulmonary rehabilitation remains a cornerstone of treatment, while adequate dietary protein intake and correction of nutritional deficiencies may further support muscle preservation and functional recovery (PMID: 41057104).

## 4. Methods for Assessing Muscle Mass

**Figure 1:**
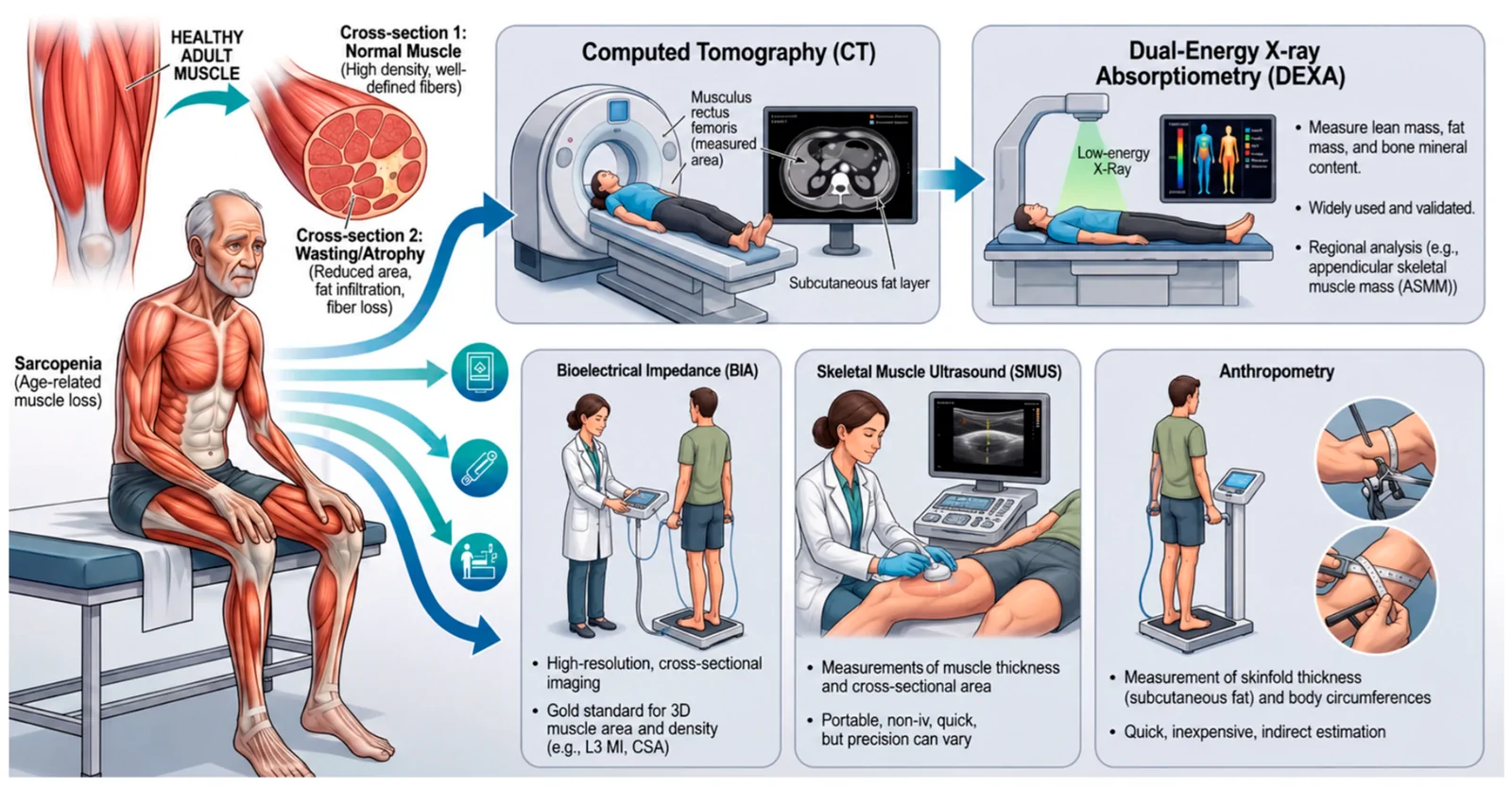
Diagnostic Modalities for the Assessment and Quantification of Muscle Mass and Sarcopenia.

A visual overview of the principal diagnostic modalities for assessing muscle mass is provided in Figure 1, which contrasts healthy muscle architecture with sarcopenic changes and illustrates the spectrum of available techniques—from advanced imaging (CT, MRI) to more accessible methods (DXA, ultrasound, BIA, and anthropometry).

Figure 1 and Table 2 provides a visual overview of the principal diagnostic modalities available for assessing muscle mass and quantifying sarcopenia. These techniques range from advanced imaging methods such as CT and MRI to more accessible tools such as DXA, ultrasound, BIA, and anthropometry, each offering distinct advantages and limitations in the evaluation of muscle quantity and quality.

### 4.1. Computed Tomography (CT)

Computed tomography is considered a highly accurate non-invasive method for assessing skeletal muscle mass [48,49,50]. However, its use in clinical practice is limited by radiation exposure, cost, complexity, and accessibility. Although CT is not routinely indicated for COPD diagnosis, it is commonly used to characterize disease phenotypes, identify coexisting lung conditions, evaluate lung volume reduction, and in lung cancer screening programs [51,52,53]. Thus, many COPD patients may already have CT imaging available without requiring additional imaging solely for muscle assessment. Measurement of skeletal muscle area (SMA) at the third lumbar vertebra (L3) provides an accurate estimate of whole-body muscle mass, but L3 is not included on standard chest CT [54]. L3 is preferred because it includes major muscles such as the psoas, paraspinal, and abdominal wall muscles [55]. Derstine et al. evaluated alternative levels from T10 to L5, confirming L3 as the preferred level, with L2, L4, L5, L1, T12, T11, and T10 as acceptable alternatives [56]. The erector spinae muscle area at T12 has also been proposed as a thoracic surrogate for low muscle mass [57,58]. CT assessment typically quantifies cross-sectional area normalized to height, yielding the skeletal muscle index (SMI) [59]. Cut-off values for low muscle mass by CT are not yet well standardized. A systematic review of 388 studies found that the most common SMI cut-points for abdominal muscles were 52–55 cm^2^/m^2^ for men and 39–41 cm^2^/m^2^ for women; for other muscle groups, cut-points are less well defined [60]. Beyond muscle quantity, CT provides information on muscle quality through measurement of muscle attenuation. Adipose tissue typically exhibits attenuation values between −190 and −30 Hounsfield units (HU), whereas skeletal muscle is generally characterized by values between −29 and +150 HU. Greater fat infiltration within muscle (myosteatosis) results in lower muscle attenuation, reflecting poorer muscle quality. Lower muscle attenuation has also been associated with impaired physical performance and adverse clinical outcomes [61,62].

### 4.2. Dual-Energy X-Ray Absorptiometry (DXA)

DXA offers a practical alternative to CT and MRI for lean mass assessment, with very low radiation exposure, good accuracy, and relative simplicity. It is generally well tolerated and avoids some of the practical limitations of MRI, such as claustrophobia and motion-related image degradation [62,63]. DXA uses a chemical-compartment approach, estimating fat mass primarily as lipid (predominantly triglycerides) and lean soft tissue as a composite compartment comprising water, protein, carbohydrates, non-fat lipids, and soft-tissue minerals [63]. In contrast, CT and MRI assess anatomical tissue compartments, with adipose tissue measurements encompassing not only adipocytes but also non-fat components such as collagenous fibers, fibroblasts, and capillaries. Consequently, DXA-derived fat mass is generally lower, whereas lean mass is higher, than corresponding CT/MRI measurements [62,64]. DXA may also be less informative for detecting subtle changes in muscle composition because it cannot directly quantify intramuscular adipose tissue or other features of muscle quality [65]. Appendicular lean mass (ALM), representing the lean mass of the arms and legs, and the appendicular lean mass index (ALMI; ALM/height^2^) can be derived from DXA. EWGSOP2 recommends cut-offs for low muscle mass of <7.0 kg/m^2^ for men and <6.0 kg/m^2^ for women [10].

### 4.3. Skeletal Muscle Ultrasound (SMUS)

Skeletal muscle ultrasound is an emerging, practical, non-invasive, and feasible method for assessing muscle quantity and quality in patients with COPD [66]. Compared with CT/MRI, ultrasound is inexpensive, portable, radiation-free, and suitable for bedside assessment, with good reproducibility and potential utility for longitudinal monitoring of muscle changes [66,67]. The quadriceps, particularly the rectus femoris, is among the most commonly studied muscle groups because of its accessibility and relevance to lower-limb function [66]. Key variables include muscle thickness, cross-sectional area, and echo intensity, allowing both quantitative and qualitative evaluation. Limitations include operator dependence and the lack of fully standardized acquisition protocols and COPD-specific cut-offs [68]. A high-frequency linear-array transducer is typically used, with the patient in the supine position and the legs relaxed; the rectus femoris is assessed at a standardized point along the thigh, with minimal probe pressure to avoid muscle compression [69]. Limitations include operator dependence and the lack of fully standardized techniques and COPD-specific cut-offs. The 2020 SARCUS update provides standardized recommendations for patient positioning, ultrasound settings, anatomical landmarks, and measurement of different muscle groups, aiming to improve the reproducibility of skeletal muscle ultrasound [70]. Deng et al. proposed sex-specific ultrasound cut-offs for sarcopenia screening in patients with COPD. In men, a rectus femoris thickness <5.22 cm yielded 76.4% sensitivity and 75.0% specificity, whereas a cross-sectional area <6.805 cm^2^ yielded 90.9% sensitivity and 67.9% specificity. In women, corresponding cut-offs were < 4.99 cm for thickness (89.5% sensitivity, 60.0% specificity) and <6.996 cm^2^ for cross-sectional area (73.7% sensitivity, 86.7% specificity) [71]. Ye et al. found that rectus femoris echo intensity was significantly increased across all GOLD stages, whereas muscle thickness and cross-sectional area decreased significantly only in GOLD III–IV, suggesting that alterations in muscle quality may precede measurable reductions in muscle size [72]. Ultrasound is particularly useful in critically ill and hospitalized patients because it is portable and can be performed at the bedside, although image acquisition and interpretation may be challenging in individuals with substantial subcutaneous adiposity or peripheral edema [73].

### 4.4. Bioelectrical Impedance Analysis (BIA)

Bioelectrical Impedance Analysis is widely used in COPD because of its non-invasive nature, portability, and low cost [73,74]. Bioelectrical impedance analysis (BIA) measures resistance and reactance in response to a low-intensity electrical current. Because fat-free tissues contain more water and electrolytes and therefore conduct electrical current more readily than adipose tissue, BIA can be used to estimate fat-free mass (FFM) through validated prediction equations. Fat-free mass index (FFMI) is calculated as FFM/height^2^ [75]. In COPD, BIA variables—particularly phase angle and bioimpedance index—have emerged as potential predictors of handgrip and respiratory muscle strength [71,76,77,78,79]. Multifrequency BIA offers advantages over single-frequency BIA by providing information on extracellular and intracellular water distribution, which may be particularly relevant in COPD patients with altered hydration status or fluid redistribution [80,81]. Phase angle and fat-free mass index (FFMI) have been associated with prognosis in COPD, although prognostic evidence is more consistent for phase angle than for FFMI [82]. However, BIA measurements are influenced by hydration status, which may be particularly relevant in COPD patients with fluid redistribution, edema, or altered volume status [83,84]. Predictive equations are often derived from healthy populations and may be less accurate in advanced COPD or cachexia. A 2025 systematic review and meta-analysis found that BIA provides moderate diagnostic accuracy and practical utility as a screening tool for sarcopenia, particularly in primary care and community settings, while highlighting the need for standardized protocols [85]. Compared with DXA, BIA shows variable agreement in sarcopenia diagnosis [86]. Despite these limitations, BIA remains valuable for initial screening, particularly where DXA or CT are unavailable. The combined use of FFMI and phase angle may provide complementary prognostic and risk-stratification information in COPD [23,86,87]. Importantly, FFMI reflects total non-adipose tissue, including skeletal muscle as well as other lean tissues and body water. In contrast, whole-body skeletal muscle mass (SMM) or appendicular skeletal muscle mass (ASMM), corresponding to skeletal muscle mass of the upper and lower limbs, are more directly related to the assessment of low muscle quantity in sarcopenia. According to the revised European consensus on the definition and diagnosis of sarcopenia, cut-off points for low muscle quantity include ASM < 20 kg in men and <15 kg in women, or an appendicular skeletal muscle mass index (ASMI), calculated as ASM divided by height squared, <7.0 kg/m^2^ in men and <5.5 kg/m^2^ in women [10]. Low muscle mass alone is not sufficient to diagnose sarcopenia and should be interpreted together with muscle strength and, where appropriate, physical performance. To date, no specific and validated cut-off values for ASM or ASMI have been established specifically for patients with COPD. Moreover, previous studies investigating sarcopenia in COPD have used heterogeneous methods for assessing muscle mass, including different BIA devices, parameters, and cut-off values.

**Table 2:** Summary of diagnostic methods for muscle wasting assessment in COPD: parameters, strengths, limitations, and clinical applicability.

Assessment Domain	Method	Parameters Measured	Strengths	Limitations	Cut-Offs in COPD	Clinical Applicability
Muscle Mass	CT	Skeletal muscle area (SMA), Skeletal muscle index (SMI), muscle attenuation	Gold standard; assesses quantity and quality (myosteatosis)	Radiation exposure; high cost; limited accessibility; L3 level not on standard chest CT	SMI: 52–55 (M), 39–41 (W) cm^2^/m^2^ [88]	Research; selected clinical cases
	MRI	Muscle volume, cross-sectional area, intramuscular fat	No radiation; excellent soft tissue contrast	High cost; limited accessibility; claustrophobia; long acquisition time	Not well established	Research; selected clinical cases
	DXA	Appendicular lean mass (ALM), ALM/height^2^	Low radiation; widely available; established cut-offs	Cannot assess muscle quality; less sensitive to small changes	ALMI < 7.0 (M), <6.0 (W) kg/m^2^ [10]	Clinical practice; research
	BIA	Fat-free mass (FFM), FFMI, phase angle	Portable; inexpensive; non-invasive	Hydration-dependent; population-specific equations needed	FFMI variable; phase angle < 4.5° [89]	Screening; clinical practice
	Ultrasound	Muscle thickness, cross-sectional area, echo intensity	Portable; inexpensive; no radiation; bedside applicability	Operator-dependent; no standardized cut-offs	Thickness: 5.22 cm (M), 4.99 cm (W); CSA: 6.8/7.0 cm^2^ [71]	Screening; ICU; research
Muscle Strength	Handgrip dynamometry	Maximal grip strength	Simple; quick; inexpensive; correlates with overall strength	Does not assess lower limb strength specifically	<27 kg (M), <16 kg (W)—EWGSOP2 [10]	Routine screening; clinical practice
	Knee extension dynamometry	Quadriceps maximal voluntary contraction	Directly assesses key muscle group in COPD; strong prognostic value	Requires specialized equipment; less standardized	QMVC < 26.2 Kg [90]	Clinical practice; research
	MIP manometry	Maximal inspiratory pressure	Assesses respiratory muscle strength; prognostic value	Effort-dependent; requires patient cooperation	MIP < 60 cmH_2_O [91]	Clinical practice; research
Physical Performance	6MWT	6 min walk distance	Integrates multiple systems; ecologically valid; prognostic	Non-specific; influenced by non-muscle factors	<350 m (risk threshold) [92]	Routine clinical practice; research
	Gait speed	Walking velocity (m/s)	Simple; rapid; reproducible; strong predictive validity	Non-specific; influenced by multiple factors	<0.8 m/s (impairment) [93]	Screening; clinical practice
	STS	Number of stands/30 s or time for 5 stands	Assesses lower limb function; correlates with quadriceps strength	Chair height standardization issues; armrest use	≤8 stands/30 s (poor performance) [94]	Clinical practice; research
	SCPT	Stair climb power (W/kg)	Specific for lower limb power; integrates with cardiopulmonary function	Safety concerns; not suitable for severe COPD	No established COPD-specific cut-off [95]	Research; selected patients
	CPET	VO_2_ peak; anaerobic threshold	Gold standard for integrated function; identifies limitation mechanism	Maximal; expensive; requires specialized equipment	<15 mL/kg/min (reduced exercise capacity) [96,97]	Research; specialized centers
	SPPB	Gait speed, chair stands, balance test	Comprehensive; validated; prognostic	Time-consuming; requires specialized training	≤8 points [98]	Research; selected clinical cases

CT = computed tomography; MRI = magnetic resonance imaging; DXA = dual-energy X-ray absorptiometry; BIA = bioelectrical impedance analysis; MIP = maximal inspiratory pressure; 6MWT = six-minute walk test; STS = sit-to-stand; SCPT = stair climb power test; CPET = cardiopulmonary exercise testing; SPPB = short physical performance battery; ICU = intensive care unit.

### 4.5. Skeletal Muscle Biopsy

Skeletal muscle biopsy provides a direct tissue-level assessment of peripheral skeletal muscle and has been extensively used in research on muscle dysfunction in COPD [99]. The vastus lateralis is the most commonly sampled muscle because of its accessibility and relevance to lower-limb function. Histological analysis can characterize muscle fiber cross-sectional area, fiber-type distribution, capillarization, and structural abnormalities, while biochemical and molecular analyses provide information on oxidative metabolism, mitochondrial function, redox status, protein metabolism, and other pathways involved in muscle dysfunction [99,100]. These tissue-level measurements have contributed substantially to the characterization of structural and metabolic abnormalities in peripheral muscle in COPD [99]. However, muscle biopsy should not be considered a clinical gold standard for diagnosing sarcopenia. The procedure is invasive, may cause discomfort and minor complications, and samples only a small portion of a single muscle; therefore, biopsy findings cannot directly represent whole-body skeletal muscle mass or fully capture regional heterogeneity [99]. Its primary value consequently lies in mechanistic and validation studies rather than routine clinical assessment. Importantly, biopsy-derived characteristics can be related to clinically relevant measures of muscle function and exercise capacity. Studies in COPD have demonstrated associations between muscle biochemical or structural characteristics and quadriceps performance, peak oxygen uptake, and physical activity, supporting the biological relevance of tissue-level abnormalities [101]. Biopsy findings may therefore complement non-invasive assessments by helping distinguish alterations in muscle quantity from qualitative abnormalities involving fiber phenotype, oxidative capacity, capillarization, and muscle metabolism. Minimally invasive microbiopsy techniques have also been validated in COPD and may provide sufficient tissue for selected biochemical and molecular analyses while reducing discomfort compared with conventional Bergström biopsy [102,103]. Nevertheless, because of its invasiveness and limited representativeness of whole-body muscle, conventional biopsy remains primarily a research tool. In clinical practice, CT/MRI, DXA, ultrasound, and BIA are preferable for longitudinal assessment, whereas muscle biopsy can provide complementary tissue-level information when mechanistic characterization or validation of non-invasive biomarkers is required.

### 4.6. Dynapenia and Dissociation Between Muscle Mass and Function

An important clinical consideration in COPD is the potential dissociation between skeletal muscle quantity and muscle function. Muscle strength may decline earlier or more rapidly than muscle mass, resulting in a dynapenic phenotype in which muscle mass is relatively preserved but strength and physical performance are substantially impaired. Consequently, a normal FFMI or appendicular lean mass does not exclude clinically relevant muscle dysfunction. In such patients, management should be guided primarily by the functional deficit rather than by muscle mass alone. Progressive resistance training should be a central component of pulmonary rehabilitation, complemented by aerobic exercise, correction of reversible contributors to weakness, optimization of COPD treatment and comorbidities, and adequate nutritional intake. Resistance training has been shown to improve muscle strength in COPD even when increases in muscle mass are limited [98,104,105]. Therefore, longitudinal assessment should include both structural measures of muscle quantity and functional measures of strength and physical performance.

## 5. Nutritional Assessment

Beyond the direct measurement of muscle mass, strength, and physical performance, evaluation of dietary intake and nutritional status represents a fundamental component of the comprehensive assessment of muscle wasting in COPD. Malnutrition and sarcopenia frequently coexist in patients with COPD, and their combination—termed malnutrition-sarcopenia syndrome—is associated with worse clinical outcomes than either condition alone.

### 5.1. Nutritional Screening Tools

Several validated instruments are available for the initial identification of patients at nutritional risk. The Mini Nutritional Assessment (MNA) and its Short-Form version (MNA-SF) have been extensively studied in COPD, identifying 22% of outpatients as malnourished and an additional 43% as at risk of malnutrition in one cohort [106]. The MNA score correlates with dyspnea severity, airflow limitation, and quality of life and predicts acute exacerbations with sub-hazard ratios of 3.08 for at-risk patients and 4.64 for malnourished patients [107]. Other widely used screening tools include the Nutritional Risk Screening 2002 (NRS-2002), the Malnutrition Universal Screening Tool (MUST), and the Patient-Generated Subjective Global Assessment (PG-SGA), each with distinct operating characteristics in COPD populations [108]. The Global Leadership Initiative on Malnutrition (GLIM) criteria have been proposed as a consensus framework for malnutrition diagnosis, with increasing evidence supporting their application in COPD [90]. The GLIM diagnostic process follows a three-step approach: (1) nutritional risk screening using validated tools such as MNA-SF or NRS-2002; (2) malnutrition diagnosis based on the presence of at least one phenotypic and one etiologic criterion; and (3) severity grading [66]. In COPD populations, evidence supports the clinical utility of GLIM criteria. A prospective cohort study of 200 stable COPD patients referred to pulmonary rehabilitation found a malnutrition prevalence of 45% according to GLIM criteria, with malnutrition associated with nearly three-fold increased mortality and hospitalization risk over 2-year follow-up [109]. Similarly, a cross-sectional study of hospitalized COPD patients reported that 45.6% met GLIM criteria for malnutrition, and the coexistence of malnutrition and sarcopenia was identified in 32.5% of patients, conferring more than twice the odds of prolonged hospital stay and 6-month readmission [110]. In elderly hospitalized COPD patients, GLIM-defined malnutrition prevalence reached 49.5% and was identified as an independent risk factor for 180-day readmission (OR = 3.184) [111]. Comparative studies have demonstrated that GLIM criteria show good concordance with other diagnostic tools. In AECOPD patients, GLIM identified 47.8% as malnourished, with good concordance with Subjective Global Assessment (SGA) (kappa = 0.533; AUC = 0.768; 95% CI 0.701–0.835) [112]. The Geriatric Nutritional Risk Index (GNRI) and Prognostic Nutritional Index (PNI) have shown high sensitivity (89.5% and 74.1%, respectively) and specificity (77.2% and 66.4%) compared with GLIM criteria, suggesting their potential as alternative screening tools in resource-limited settings [113]. However, prospective validation studies examining GLIM’s predictive validity for patient-centered outcomes in COPD remain limited, and the optimal screening tool to precede GLIM diagnosis in COPD outpatients requires further standardization. Nevertheless, current evidence supports the integration of GLIM criteria into comprehensive nutritional assessment for COPD patients, particularly in hospital and rehabilitation settings [90,111].

### 5.2. Dietary Intake Assessment

Effective integration of nutritionists into the diagnostic workflow for muscle wasting in COPD requires a structured, multidisciplinary approach, as emphasized by recent consensus documents [90]. Nutritionists should be involved in initial screening and risk stratification, administering validated screening tools during routine COPD reviews, with clear referral criteria including BMI below 21 kg/m^2^, unintentional weight loss exceeding 5% over 6 months, an MNA score below 17, or an NRS-2002 score of 3 or higher [114]. Subsequently, comprehensive nutritional assessment should include detailed evaluation of dietary intake, typically using food frequency questionnaires, 24 h dietary recalls, or food diaries [115,116]. These approaches provide information on macro- and micronutrient consumption that may contribute to muscle depletion. Nutritionists should interpret body composition data from BIA or DXA in the context of dietary intake and clinical status, identifying specific deficits such as inadequate protein intake or distribution, leucine insufficiency, or vitamin D deficiency, and should apply GLIM criteria when appropriate to confirm malnutrition diagnosis [90]. Emerging evidence suggests that the Dietary Inflammatory Index (DII), a measure of the overall inflammatory potential of the diet, is associated with sarcopenia prevalence in COPD patients; higher DII scores, indicating a more pro-inflammatory dietary pattern, correlate with increased sarcopenia risk and all-cause mortality [117]. Inadequate intake of protein, vitamin D, antioxidants, and omega-3 fatty acids has been identified as a potentially modifiable contributor to muscle deterioration in COPD, potentially promoting anabolic resistance and muscle catabolism [68]. At the intervention and monitoring stage, nutritionists should develop individualized dietary prescriptions including energy targets of 25–30 kcal/kg/day, protein targets of 1.2–1.5 g/kg/day, adequate protein distribution across meals with approximately 2.5–3.0 g leucine per meal, and targeted micronutrient supplementation, where indicated [118,119]. Coordination with physiotherapists may also help integrate protein intake with exercise sessions, followed by regular nutritional and functional reassessment. Finally, multidisciplinary team integration requires regular case conferences to review complex cases and coordinate care plans, with nutritionists participating in pulmonary rehabilitation programs [118]. The integration of dietary intake assessment into routine COPD care is therefore important for identifying modifiable nutritional deficits and guiding individualized interventions.

### 5.3. Specific Nutrient Assessment

Several key nutrients warrant particular attention in the evaluation of COPD-related muscle wasting. Protein intake is critical for maintaining muscle mass, with recommendations emphasizing adequate intake of high-quality, leucine-rich sources to support muscle protein synthesis [119]. A systematic review and meta-analysis of 29 randomized controlled trials demonstrated that nutrient supplements significantly improve body weight (mean difference 1.33 kg), fat-free mass index (0.74 kg/m^2^), and 6 min walk distance (19.43 m) in patients with COPD [120]. High-protein diets combined with physical exercise have also shown clinically relevant effects on walking distance during pulmonary rehabilitation [121]. Vitamin D deficiency is more prevalent in COPD patients compared with controls and has been linked to lower lung function, muscle atrophy, and impaired musculoskeletal function [122]. Vitamin D deficiency may affect inflammation, oxidative stress, and mitochondrial function, potentially contributing to skeletal muscle atrophy [122]. A randomized controlled trial demonstrated that 16 weeks of whey protein combined with vitamin D supplementation improved handgrip strength, gait speed, and physical performance in COPD patients [123]. Omega-3 polyunsaturated fatty acid supplementation has been shown to induce a shift toward positive daily protein homeostasis in a dose-dependent manner, with daily doses up to 3.5 g of EPA and DHA being well tolerated and associated with protein gain [124]. Integrating nutritional assessment into the diagnostic workup of muscle wasting in COPD is therefore important. Patients identified as malnourished or at nutritional risk should receive targeted dietary counseling and, when appropriate, oral nutritional supplementation, preferably in conjunction with exercise training and pulmonary rehabilitation [90].

### 5.4. Automating Sarcopenia and Malnutrition Pathways: Technical Platforms and Workflows

The automation of clinical care pathways for the management of sarcopenia and malnutrition represents an emerging shift from manual, reactive documentation toward more proactive, data-driven clinical interventions. By integrating software platforms, machine learning, and automated EHR workflows, healthcare systems may help reduce diagnostic delays, alleviate clinician workload, and support the delivery of nutritional and physical rehabilitation strategies [125]. At the core of these systems, EHR integration modules connect a healthcare organization’s digital health record with other software, databases, or devices, allowing patient data to flow automatically without manual entry. HL7 FHIR (Fast Healthcare Interoperability Resources) APIs (Application Programming Interfaces) can retrieve structured information, including serial body weight, BMI, laboratory results, and demographic characteristics, while also incorporating unstructured clinical notes [126]. Complementing this infrastructure, Clinical Decision Support Systems (CDSS) integrated into EHR platforms can process incoming data and dynamically calculate risk scores without requiring manual assessment [127]. Advanced AI and diagnostic software may further support precision assessment. Computer vision engines based on deep learning architectures, such as U-Net, can analyze routine CT, MRI, or ultrasound images obtained for other clinical indications to enable opportunistic assessment of skeletal muscle index (SMI) and cross-sectional muscle area [128]. Similarly, machine-learning-based risk predictors can integrate nonlinear variables, including comorbidity indices, age, medication profiles such as systemic corticosteroid exposure, and gastrointestinal symptoms, to generate patient-specific risk scores [129]. Given the heterogeneous clinical phenotypes of COPD, ranging from underweight patients with severe muscle wasting to individuals with sarcopenic obesity, AI-driven CDSS may eventually facilitate more personalized nutritional screening and intervention. By continuously integrating multidimensional EHR data, such systems could potentially identify early or otherwise unrecognized muscle loss, including in normal-weight or obese patients, and support phenotype-specific nutritional interventions aligned with metabolic status and pulmonary rehabilitation goals. However, these applications remain emerging and require prospective validation, attention to data quality and algorithmic transparency, and demonstration of clinical benefit before routine implementation. Finally, connected devices and IoT-enabled ecosystems may extend monitoring beyond the clinical setting. Wireless dynamometers for handgrip strength, skinfold calipers, and smart scales incorporating bioelectrical impedance analysis (BIA) can potentially be connected to digital health records through Bluetooth-enabled systems [128]. Patient-facing telemedicine platforms and mobile health applications can additionally capture patient-reported outcomes, including remote BAPEN Self-Screening Tool or R-MAPP assessments, together with accelerometer-derived activity data from consumer wearables, potentially enabling remote monitoring of physical function and frailty [130].

### 5.5. Human, Cultural and Organizational Barriers to Implementing AI-Driven Nutrition Systems in COPD

When implementing AI/ML-driven Clinical Decision Support Systems (CDSS) for nutrition management in COPD patients, significant organizational and doctor-patient communication barriers may arise across four key dimensions: coherence, cognitive participation, collective action, and reflexive monitoring. Reported obstacles include privacy concerns, blurred boundaries regarding authority, technical anxiety, communication difficulties, and additional workload associated with system use [131]. Within organizational and clinical cultures, a critical challenge is the lack of algorithmic transparency, which may leave healthcare providers uncertain about whether to rely on or challenge AI-generated outputs. Older COPD patients may also experience a digital divide, while severe physical symptoms such as dyspnoea may make repetitive daily tasks—such as food logging through rigid digital interfaces—particularly burdensome [132]. Furthermore, cultural and social dynamics may limit the applicability of standardized AI nutrition models when they fail to account for regional, ethnic, or traditional family eating habits. Uncertainty regarding clinical accountability and socioeconomic disparities may also restrict access to recommended foods or interventions [133]. These considerations highlight the need for AI-supported nutritional systems to complement rather than replace professional judgment, patient preferences, and individualized multidisciplinary care.

## 6. Methods for Assessing Muscle Strength

**Figure 2:**
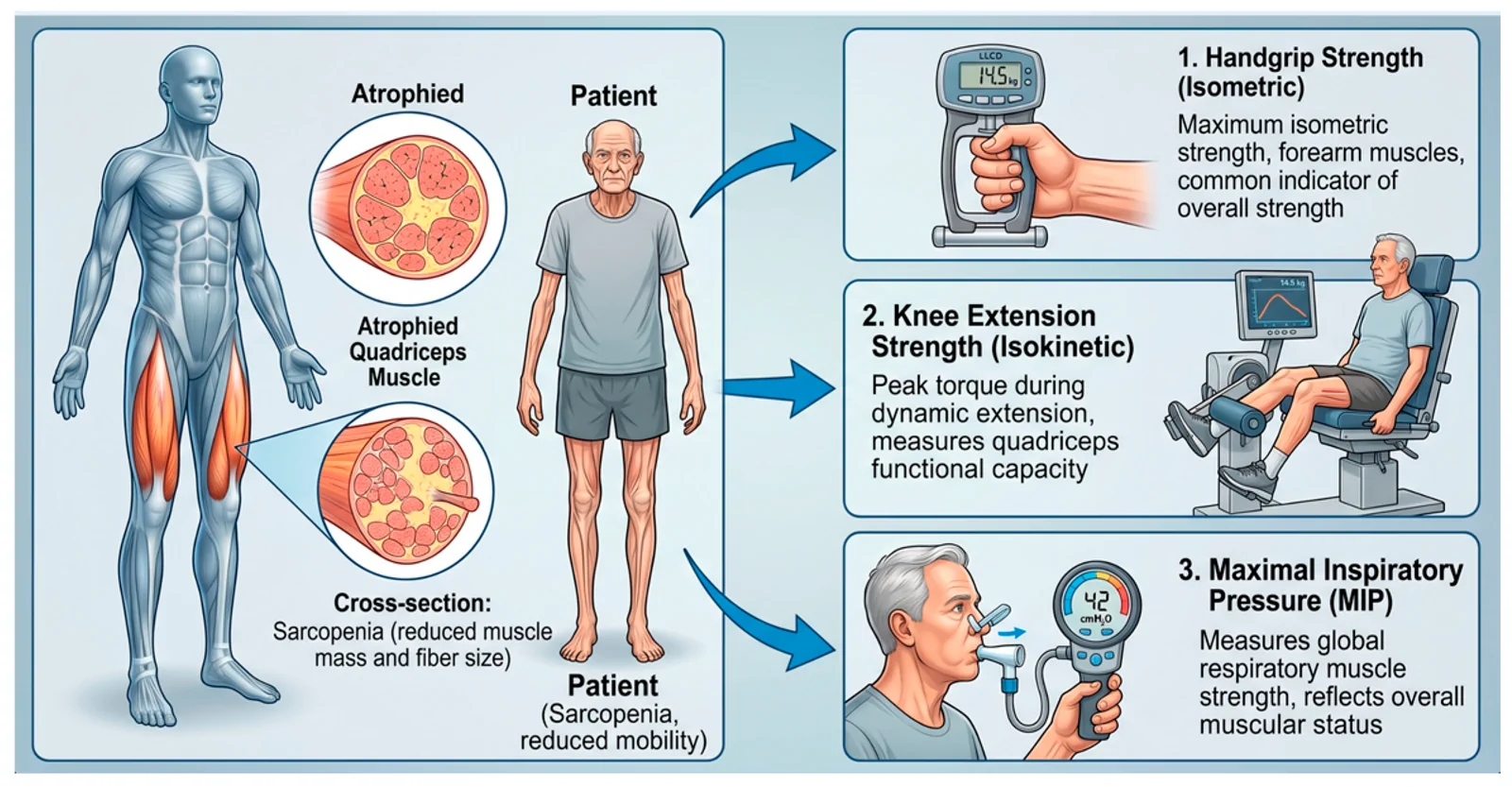
Multidimensional Assessment of Muscle Strength in Sarcopenia.

Figure 2 summarizes the multidimensional approach to muscle strength assessment in sarcopenia, encompassing handgrip dynamometry, knee extension dynamometry, and maximal inspiratory pressure, alongside a cross-sectional depiction of quadriceps atrophy.

### 6.1. Handgrip Strength

Handgrip strength measured by dynamometry is the most widely used method for assessing overall muscle strength in clinical practice. It is simple, quick, inexpensive, and highly reproducible, making it suitable for routine screening [10]. Handgrip strength correlates with lower limb strength and predicts disability, hospitalization, and mortality [134]. EWGSOP2 identifies low handgrip strength as the primary indicator of probable sarcopenia, recommending cut-offs of <27 kg for men and <16 kg for women [8]. AWGS 2019 proposes different thresholds (<28 kg and <18 kg) based on Asian population data [12], while SDOC suggests higher cut-offs (<35.5 kg and <20 kg), reflecting its function-oriented approach [135]. In COPD, handgrip strength correlates with quadriceps strength, exercise capacity, and quality of life [104]. However, it cannot differentiate between muscle loss and neurological or other causes of weakness, nor does it provide information about muscle quality or mass [136].

### 6.2. Knee Extension Strength

Knee extension strength, measured using a handheld or fixed dynamometer, specifically assesses quadriceps function—the muscle group strongly affected in COPD-related wasting and a key determinant of mobility and exercise capacity [137]. Quadriceps strength correlates with CT-assessed muscle cross-sectional area and has been reported to be a predictor of mortality in COPD [138]. Measurement protocols generally involve isometric maximal voluntary contraction with the knee flexed at 90°. Cut-off values are less standardized than for handgrip, with no universally accepted absolute threshold; functionally relevant COPD-specific thresholds include values below 8.30 kg/m^2^ in men and 5.99 kg/m^2^ in women when normalized to height squared [139]. Limitations include the need for specialized equipment, standardization difficulties, and patient comfort issues. Nevertheless, knee extension strength provides a more direct assessment of the muscle group most relevant to mobility and exercise capacity in COPD.

### 6.3. Maximal Inspiratory Pressure (MIP)

Maximal inspiratory pressure reflects respiratory muscle strength, primarily diaphragmatic function. In COPD, respiratory muscle dysfunction contributes to dyspnoea, exercise limitation, and hypercapnic failure. MIP is measured using a manometer during maximal inspiratory effort, usually from residual volume [140]. There are no universally accepted absolute cut-off values for MIP, although values ≤ 55 cmH_2_O have been frequently reported as functionally or prognostically relevant in COPD cohorts [140]. MIP correlates with peripheral muscle strength and exercise capacity in COPD, and lower MIP is associated with increased mortality and hospitalization [141]. However, MIP does not directly assess peripheral muscle mass or strength and therefore should be considered complementary rather than a comprehensive measure of sarcopenia in COPD.

Figure 2 illustrates the principal methods for evaluating muscle strength: handgrip dynamometry (isometric assessment of forearm muscles), knee extension dynamometry (assessment of quadriceps functional capacity), and maximal inspiratory pressure (MIP) manometry for respiratory muscle strength. The cross-sectional image depicts sarcopenic quadriceps atrophy with reduced muscle mass and fiber size. MIP = maximal inspiratory pressure.

## 7. Muscle Endurance and Functional Performance Testing

### 7.1. Six-Minute Walk Test (6MWT)

The 6MWT is a submaximal exercise test widely used to assess functional exercise capacity in COPD, measuring the distance walked in six minutes according to standardized ATS/ERS guidelines [142]. The test reflects integrated cardiovascular, respiratory, and skeletal muscle function, making it particularly relevant for evaluating functional consequences of muscle wasting [142,143]. The 6MWD correlates moderately to strongly with quadriceps strength and endurance, as peripheral muscle dysfunction is an important determinant of exercise intolerance beyond lung function impairment alone. In COPD, values below approximately 350 m are associated with increased risk of hospitalization and mortality [143]. The test is sensitive to changes following pulmonary rehabilitation, with improvements in 6MWD paralleling improvements in exercise capacity and functional status [142,143]. Strengths include simplicity, low cost, minimal equipment requirements, good reproducibility, and excellent ecological validity, as it reflects daily walking activity [142,143]. Unlike maximal tests, the 6MWT is generally well tolerated by patients with severe COPD. Limitations include influence by multiple factors beyond muscle function, including dyspnoea, cardiovascular comorbidities, motivation, and learning effects, as well as its lack of specificity for diagnosing sarcopenia and inability to provide information about isolated muscle mass or specific muscle-group function [142,143]. The 6MWT is therefore best used as a measure of functional impairment, complemented by direct measures of muscle mass and strength [143].

### 7.2. Gait Speed

Gait speed, measured as the time required to walk a predetermined distance, typically 4 or 6 m, is a simple, rapid, and highly reproducible measure of physical performance and has been proposed as a “vital sign” of functional status in older adults and COPD patients [144]. Slow gait speed (<0.8 m/s) is associated with muscle weakness, reduced physical performance, and increased risk of disability, hospitalization, and mortality [23]. Gait speed reflects integrated physiological function, with skeletal muscle strength and endurance being important determinants. In COPD, gait speed decreases with increasing disease severity, and values below 0.8 m/s are associated with poorer functional capacity and adverse outcomes [93,145]. Gait speed is responsive to pulmonary rehabilitation, with changes of approximately 0.1 m/s considered clinically meaningful [87]. Strengths include exceptional simplicity, very low cost, minimal patient burden, high reproducibility, and applicability across COPD severity grades [144,145]. Limitations include the influence of non-muscular factors such as balance disorders, arthritis, cardiovascular disease, neurological conditions, and fear of falling, as well as the inability to differentiate muscle mass loss from other causes of impaired mobility [144,145]. Gait speed is therefore particularly useful as a screening and longitudinal monitoring measure and should be interpreted together with muscle mass and strength assessments [93].

### 7.3. Sit-to-Stand Test (STS)

The STS, particularly the 30 s or 5-repetition version, assesses lower-extremity strength, power, and endurance by measuring the number of stands from a chair within a specified time or the time required to complete five stands [146]. The test primarily loads the quadriceps, gluteal, and hip flexor muscles, making it relevant for evaluating peripheral muscle dysfunction in COPD [146]. STS performance correlates with quadriceps strength and exercise capacity and is associated with functional status and prognosis in COPD [146,147]. A change of ≥2 repetitions in the 30 s STS is considered clinically meaningful [147]. Strengths include good reproducibility, minimal training requirements, and predictive value for functional outcomes and mortality. The test integrates muscle strength, power, and endurance, offering more comprehensive information than isolated strength measurements. Limitations include dependence on balance, joint function, cardiovascular capacity, and test protocol, as well as the influence of chair height and armrest use [146]. The STS is best used as a functional measure of lower-limb performance, combined with direct muscle mass assessment and other functional tests.

### 7.4. Stair Climbing Power Test (SCPT)

The SCPT requires patients to ascend a flight of stairs at normal or maximal pace, with power calculated from body mass, vertical height, and ascent time [95]. The test imposes a substantial load on the lower-limb muscles, particularly the quadriceps, making it relevant for evaluating muscle power and functional performance in COPD [95]. In COPD, SCPT performance is associated with quadriceps strength and functional exercise capacity, although it should not be considered a direct surrogate of muscle strength [95]. Strengths include integration of muscular and cardiopulmonary function and minimal equipment requirements [95]. Limitations include cardiopulmonary limitations, difficulty in patients with severe disease or balance impairment, protocol standardization issues, and safety concerns. The SCPT is therefore most appropriate for selected patients able to safely perform stair climbing and should be complemented by direct assessments of muscle strength and mass.

### 7.5. Cardiopulmonary Exercise Test (CPET) and VO_2_ Peak

CPET is the reference method for assessing integrated cardiopulmonary and musculoskeletal function during maximal exercise, with VO_2_ peak representing the maximum rate of oxygen uptake [87]. VO_2_ peak is determined by cardiac output and peripheral oxygen extraction, with skeletal muscle function being an important determinant in COPD [148]. In COPD patients, VO_2_ peak is typically reduced, with values below approximately 15 mL/kg/min associated with increased mortality risk [148,149]. Peripheral muscle dysfunction contributes substantially to exercise limitation and VO_2_ peak impairment independently of lung function [149]. VO_2_ peak correlates with quadriceps strength, fat-free mass index, and muscle oxidative capacity [148]. Strengths include comprehensive assessment of integrated physiological function, the ability to identify primary limiting factors such as ventilatory, cardiovascular, or muscular limitations, and strong prognostic value [148,149]. Limitations include the need for maximal effort, dependence on patient motivation, influence by multiple systems beyond muscle function, expensive equipment, specialized expertise, medical supervision, and safety considerations [148]. CPET is therefore best used as a comprehensive assessment tool in specialized centers for patients with unclear exercise limitation mechanisms or in selected patients undergoing major interventions, rather than as a routine screening test for muscle wasting. For this purpose, CPET should be complemented by direct measures of muscle mass and isolated muscle strength.

## 8. Future Directions

**Figure 3:**
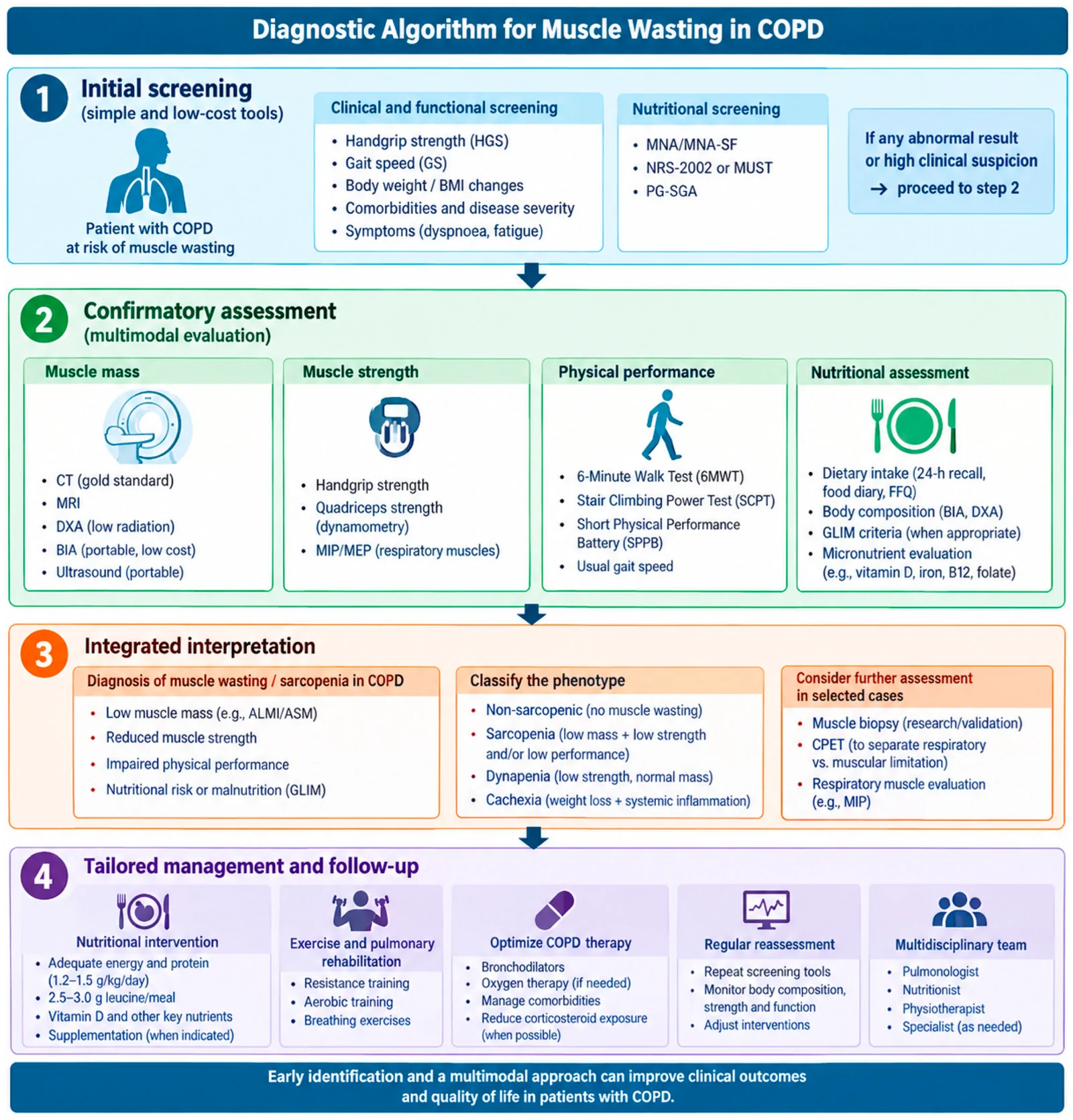
Proposed diagnostic algorithm for the assessment of muscle wasting in COPD.

Several areas require further investigation to improve the diagnosis and management of muscle wasting in COPD. First, standardized diagnostic criteria specific to COPD are needed. Current sarcopenia definitions were developed primarily for community-dwelling older adults and may not fully capture the distinct pathophysiology of muscle wasting in COPD, where systemic inflammation, hypoxia, corticosteroid exposure, nutritional impairment, and altered physical activity contribute to muscle dysfunction. Integration of multiple diagnostic modalities into practical clinical algorithms is therefore an important priority. Evidence-based algorithms combining simple screening tools with targeted assessments in patients at increased risk could optimize resource utilization while maintaining diagnostic accuracy. Based on the evidence reviewed, we propose a practical diagnostic algorithm integrating initial clinical and nutritional screening with targeted assessment of muscle mass, strength, and physical performance, followed by integrated phenotypic classification and individualized management (Figure 3). Machine-learning approaches may eventually help identify informative combinations of clinical, functional, imaging, and nutritional variables, although such models require external validation and demonstration of clinical utility before implementation. Longitudinal studies are also needed to better define the trajectory of muscle decline in COPD and identify modifiable risk factors. Particular attention should be given to patients in whom muscle strength or quality deteriorates before substantial changes in muscle quantity become apparent. Finally, elucidation of the underlying pathophysiological mechanisms remains a priority. Although systemic inflammation and oxidative stress are recognized contributors, the molecular pathways linking COPD to muscle depletion remain incompletely understood. Further investigation of mitochondrial dysfunction, anabolic resistance, altered protein turnover, endocrine signaling, hypoxia, and nutritional status may identify biomarkers capable of detecting early muscle dysfunction and informing personalized interventions.

The algorithm integrates an initial low-cost screening phase with targeted confirmatory assessments and subsequent integrated clinical interpretation. Patients at risk undergo clinical/functional and nutritional screening, followed, when abnormalities or clinical suspicion are present, by assessment of muscle mass, muscle strength, physical performance, and nutritional status using complementary methods. Findings are then integrated to identify muscle wasting/sarcopenia and distinguish relevant phenotypes, including sarcopenia, dynapenia, and cachexia, while respiratory muscle dysfunction and other causes of exercise limitation may be further investigated in selected patients. The final step emphasizes individualized nutritional intervention, exercise and pulmonary rehabilitation, optimization of COPD management, and regular reassessment within a multidisciplinary care pathway. This algorithm represents a proposed evidence-informed framework and requires prospective validation before implementation as a standardized clinical pathway.

## 9. Conclusions

Skeletal muscle wasting is a frequent and clinically consequential comorbidity in COPD, affecting approximately 15% to more than 50% of patients depending on the diagnostic criteria and population studied. Its clinical importance is underscored by associations with reduced exercise tolerance, impaired quality of life, increased healthcare utilization, and elevated mortality risk. Despite this prognostic relevance, the optimal approach for diagnosing muscle wasting in COPD remains debated, reflecting patient heterogeneity and the absence of standardized criteria specific to this condition.

This review has critically examined the strengths and limitations of currently available diagnostic modalities. Imaging techniques, particularly CT and MRI, provide accurate assessment of muscle quantity and quality but are constrained by cost, radiation exposure in the case of CT, and limited accessibility. DXA provides a practical alternative with low radiation exposure but does not adequately characterize muscle quality. BIA and ultrasound are portable and inexpensive, yet their accuracy can be influenced by hydration status, operator dependence, and the lack of standardized COPD-specific cut-offs. Functional performance tests—including gait speed, the 6 min walk test, and the sit-to-stand test—provide clinically meaningful assessments of physical performance but do not directly quantify muscle mass. Each modality therefore provides complementary information, and no single test can comprehensively capture the multifaceted nature of muscle wasting.

This multimodal principle also applies to nutritional evaluation. Nutritional status should not be considered separately from muscle assessment but as an integral component of the clinical characterization of COPD-associated muscle wasting. Screening tools, dietary assessment, body composition measurements, and GLIM criteria can identify potentially modifiable nutritional abnormalities that may contribute to muscle loss and influence response to rehabilitation [150]. The marked variability in sarcopenia prevalence estimates across COPD studies reflects differences in patient populations, diagnostic definitions, and assessment methods. This underscores the need for standardized, COPD-specific diagnostic criteria integrating validated measures of muscle mass, strength, physical performance, and nutritional status.

Future research should prioritize practical clinical algorithms combining simple screening tools with targeted confirmatory assessments [151], establish prognostic thresholds tailored to COPD, and determine whether early identification and treatment of muscle dysfunction improve patient-centered outcomes. Notwithstanding current uncertainties, several practical recommendations can be offered. Patients with COPD, particularly those with advanced disease, frequent exacerbations, reduced exercise capacity, or nutritional impairment, should undergo routine screening for muscle dysfunction using simple measures such as gait speed or handgrip strength [152], together with validated nutritional screening tools such as the MNA or MUST. Patients identified as being at risk should receive more comprehensive evaluation, including body composition analysis, functional testing, and detailed dietary assessment, with particular attention to protein and relevant micronutrient intake.

Nutritional assessment and intervention should therefore be systematically integrated into COPD care. Adequate protein intake and correction of documented nutritional deficiencies may support muscle preservation, while targeted oral nutritional supplementation can be considered in patients with malnutrition or specific nutritional deficits. Leucine-rich protein supplementation combined with vitamin D may be considered in selected patients, although nutritional interventions should be individualized according to clinical status and the available evidence. Finally, pulmonary rehabilitation, with particular emphasis on resistance exercise training, should be offered to patients with functional impairment, given its established benefits for muscle function, exercise capacity, and quality of life [153].

Ultimately, recognizing and addressing muscle wasting as an integral component of COPD management—rather than as an ancillary complication—may help improve functional outcomes and reduce its clinical burden. Achieving this goal will require continued research, standardized diagnostic approaches, multidisciplinary care, and implementation strategies capable of translating advances in muscle and nutritional assessment into meaningful clinical practice.

## Figures and Tables

**Table 1:** Comparison of major sarcopenia consensus definitions, diagnostic criteria, and recommended cut-off values.

Consensus Group	Definition	Key Diagnostic Criteria	Cut-Off Values
EWGSOP 2010 [14]	Low muscle mass + low muscle strength OR low physical performance	DXA or BIA muscle mass; handgrip strength; gait speed or SPPB	DXA ALMI: <7.26 kg/m^2^ (M), <5.5 kg/m^2^ (W); Handgrip: <30 kg (M), <20 kg (W); SPPB ≤ 8
EWGSOP2 2019 [10]	Low muscle strength first (probable); confirmed with low mass/quality; severe with poor performance	Handgrip strength; muscle mass by DXA/BIA/CT; physical performance	Handgrip: <27 kg (M), <16 kg (W); DXA ALMI: <7.0 kg/m^2^ (M), <6.0 kg/m^2^ (W)
AWGS 2019 [12]	Age-related muscle loss + reduced strength AND/OR impaired performance	Handgrip strength; gait speed; 5-time chair stand; SPPB; DXA/BIA	Handgrip: <28 kg (M), <18 kg (W); Gait speed < 1.0 m/s; DXA ALMI: <7.0 kg/m^2^ (M), <5.4 kg/m^2^ (W); BIA: <7.0 kg/m^2^ (M), <5.7 kg/m^2^ (W)
IWGS [26]	Reduced muscle mass + impaired physical performance	Gait speed; DXA appendicular lean mass	Gait speed < 1.0 m/s; Low ALM by DXA
FNIH [27]	Gait speed, handgrip strength, DXA lean mass	Gait speed; handgrip; ALM/BMI ratio	Gait speed < 0.8 m/s; Handgrip: <26 kg (M), <16 kg (W); ALM/BMI: <0.789 (M), <0.512 (W)
SDOC [11]	Low muscle strength + slow gait speed ONLY	Handgrip strength; gait speed	Handgrip: <35.5 kg (M), <20 kg (W); Gait speed < 0.8 m/s

EWGSOP—European Working Group on Sarcopenia in Older People; AWGS—Asian Working Group for Sarcopenia; IWGS—International Working Group on Sarcopenia; FNIH—Foundation for the National Institutes of Health; SDOC—Society for Sarcopenia, Cachexia and Wasting Disease; DXA—Dual-Energy X-ray Absorptiometry; BIA—Bioelectrical Impedance Analysis; Computed Tomography; ALMI—Appendicular Lean Mass Index; ALM—Appendicular Lean Mass; BMI—Body Mass Index; SPPB—Short Physical Performance Battery; M: Male; W: Female; kg/m^2^: kilograms per square meter; m/s: meters per second.

## Data Availability

No new data were created or analyzed in this study. Data sharing is not applicable to this article.
